# Assessing parents’ and children’s psychological well-being and its associated factors during the COVID-19 lockdown in a Tunisian-North African population

**DOI:** 10.1186/s12889-023-17206-1

**Published:** 2023-11-17

**Authors:** Asma Guedria, Hela Slama, Manel Ben Fredj, Shayma Miladi, Hamdi El Kefi, Syrine Gatti, Abdelaziz Oumaya

**Affiliations:** 1 University of Monastir, Monastir, Tunisia; 2 Department of Child and Adolescent Psychiatry, Fattouma Bourguiba University Hospital, Monastir, Tunisia; 3 Military Hospital of Tunis, Tunis, Tunisia; 4 Department of Epidemiology and Preventive Medicine, Fattouma Bourguiba University, Hospital, University of Monastir, Monastir, Tunisia; 5 Department of Community Medicine, Fattouma Bourguiba University Hospital, Monastir, Tunisia; 6 Faculty of Medicine of Tunis, University of Tunis El Manar, Tunis, Tunisia

**Keywords:** Mental health, Anxiety, COVID-19, Family, Parents

## Abstract

**Background:**

The rapidly emerging Coronavirus infectious disease 2019 (COVID-19) has spread around the world yielding in significant changes in almost every aspect of daily life. While primary research of the epidemic COVID-19 has focused on the psychological impact on the general population and health professionals, no survey of the pandemic-resulting containment on parents and their children has been yet addressed in the Tunisian-North African population. This study aimed to assess the psychological profile of parents and youth in Tunisia during a period of COVID 19 lockdown, and to identify associated factors to parental anxiety symptoms.

**Methods:**

This is an analytical cross-sectional study composed of a total of 538 adults including 464 mothers and 74 fathers. Parents of children aged less than 18 years completed an online survey (Google Forms) on the Psychological Impact of Confinement which includes the Generalized Anxiety Scale (GAD-7), the Parental Burnout Assessment (PBA), and the infantile trait-anxiety scale. The survey was initiated in May 2020 on a population of the Tunisian-North African citizens and lasted for more than 6 weeks.

**Results:**

The median GAD-7 score was 11 corresponding to a moderate anxiety with 27.8% reporting severe anxiety. The median PBA score was 31 corresponding to a moderate risk of burnout, with 19.5% being affected. The children's anxiety scores were associated with their parents' anxiety ratings with 24% of the children reported signs of moderate anxiety.

**Conclusions:**

The COVID-19 pandemic affected parental and their children’ psychological behavior due to a direct social isolation and distancing. Pediatricians need to be alerted on this issue and future measures are essential to avoid parental emotional burnout and anxiety disorder in similar situations.

## Background

In December 2019, an outbreak of coronavirus disease 2019 (COVID-19) occurred in Wuhan, Hubei Province of China, which spread rapidly throughout the country and subsequently around the world. On March the 2^nd^, Tunisia confirmed the first SARS-COV-2 positive case. The victim being a 40 years-old male, who fled from Italy. The Tunisian government introduced progressive containment measures a week later to prevent contagion of the infectious disease. Since spreading of the pandemic throughout the country, Tunisian families were forced to carry out massive containment and mandatory quarantine three weeks later to contain further spread of the virus as the threat of a pandemic loomed. The living conditions of families changed suddenly and profoundly since that date as the country deployed multiple measures. Kindergartens and schools were temporarily closed, social contacts were severely restricted and leisure activities outside the home were cancelled. Parents were encouraged to support their children in home schooling. The economic situation was worsened. The COVID-19 pandemic triggered the largest global economic crisis in more than a century resulting in 7% drop in global commercial commerce in 2020. This situation resulted not only on heavy financial-related strains [1] but was certainly associated with distress, mental health problems and multiple violence cases in families. Previous study from the pandemic disasters evidenced posttraumatic stress disorder of children and their parents with varying disease-containment experiences [2]. As Masten and Obradovic [3] wrote about the pandemic illness, "families often infect each other before a person is diagnosed; they also infect each other through fear". Recent studies evidenced different levels of psychological distress among people exposed to the COVID-19 epidemic, which triggered a wide variety of psychological problems such as panic disorder, anxiety and depression [4–7]. Although some studies were made available on the impact of the pandemic on the public, including patients, medical staff, children, and elderly people [8, 9], little is known about the consequences of the pandemic disaster on family psychological and emotional health. It is hypothesized that the COVID-19 epidemic could increase parents' mental disorders, particularly anxiety at both the individual and dyadic levels, with a negative impact on children's emotional and behavioral well-being.

The objective of this study was to assess the psychological impact including anxiety and parental burnout of the COVID-19 pandemic on a Tunisian-North African population composed of parents and youth at the time when the entire country was in a state of general stalemate. We also determined independently associated factors with parental anxiety symptoms.

## Methods

### Study design and population

This is an analytical cross sectional study based on an online questionnaire. Participants completed the survey via a cloud-based questionnaire (Google Forms). The inclusion criteria of this study were: being parent of a child under 18 years old, having access to the internet and being a citizen of Tunisia. Given that no updated national list of Tunisian citizens with their contact information, it wasn't feasible to randomly select participants. As a result we used the snowball sampling method to gather participants. The required sample size was determined using the following formula: *n* = [(Zα/2) 2 × p x (1-p)]/i2. We considered a parental anxiety rate (p) of 24% [10], with a precision level (i) of 5%, a 5% error (α), and accounted for a 25% loss. This calculation led to a minimum required sample size of 350 participants.

This study was shared on Facebook for a limited time from the beginning of May to June 3^rd^ of 2020. It should be mentioned that Facebook represents the most popular social media in Tunisia [11]. Therefore, we also paid promoted Facebook ads to motivate parents to participate for the survey.

### Questionnaire

The survey consisted of 74 questions divided in 4 categories and lasts for approximately 15 min in total to complete. Both parent-related and child-related questionnaires were completed by the parent. The demographic characteristics included gender, age, occupation, educational and socio-economic levels, parental situation, place and type of residence, number of children and their ages, and their personal somatic and psychiatric history. The Arabic version of the Generalized Anxiety Disorder Scale (GAD-7) was employed to assess the anxiety symptoms of parents. The GAD-7 is a self-report questionnaire that screens and measures severity of generalized anxiety disorder. Participants rated seven items according to the frequency of symptoms in the past two weeks on a four-point scale from 0 (not at all) to 3 (nearly every day). Total score ranges from 0 to 21, with highest scores indicating great severity of anxiety symptoms. The presence of anxiety symptoms was defined as a total score of ≥ 5 points. The GAD-7 is a well-validated screening instrument which has demonstrated excellent internal consistency (Cronbach’s α = 0.911) [12].

Parental Burnout Assessment (PBA) was conducted in the Arabic version to assess parental burnout in the context of the current COVID-19 pandemic [13]. PBA is a 23‐item questionnaire assessing four distinct core dimensions of parental burnout: 1) exhaustion in one’s parental role, 2) contrast with previous parental self, 3) feelings of being fed up with one’s parental role, and 4) emotional distancing from one’s children. The original items were rated on a seven‐point Likert scale. It was simplified to a five-point (0 = never, 4 = daily). The original English and French versions of the PBA were shown to demonstrate good internal consistency, with Cronbach’s alpha reliabilities being 0.93, 0.93, 0.90, and 0.81 for the four subscales, respectively, and the correlations between the four factors varying from 0.66 (exhaustion and emotional distancing) to 0.78 (contrast with previous parental self and feelings of being fed up as a parent [14]. The presence of burnout was defined as a total score of 51.

The psychological impact of COVID-19 among children was investigated using a parent report form of the infantile trait-anxiety scale [15, 16]. It is a validated questionnaire elaborated in the Arabic Tunisian dialect to assess symptoms of anxiety disorders in children, with Cronbach’s alpha at 0.93, sensibility at 82.5% and specificity at 0.97%. It explores psychological, behavioral, and somatic dimensions of anxiety. The original 34-item scale was adjusted to a 32-item according to the current context. Participants rated each item according to the frequency of symptoms over the past 2 weeks on a four-point scale from 0 (not at all) to 3 (always). Total scores ranged from 0 to 96, with the highest score indicating greater severity of anxiety. Scores were considered normal (0–20) or indicative of mild (21–43), moderate (44–66), or severe (67–96) psychological impact.

Children’s eating disturbance was assessed using questions with Likert scale of four points (no, sometimes, often, always). These questions investigated whether children are more or less than their usual intake, and whether they had special customs or practices related to food. To investigate children’s sleep disturbance, parents were asked about children’ sleep patterns, difficulties falling asleep, and dream experiences.

In addition, we constructed additional items to explore cyber addiction. Three questions inquired the usage of electronic devices and its impact on children. The screen time duration and whether children experienced resistance or negative emotions when asked to decrease screen time. The perceived impact of electronic device usage on children's eating habits, sleep patterns, and interpersonal relationships. Classification for screen time duration were: less than 1 h, between 1–2 h, between 2–4 h and more than 4 h. Other questions were conducted with the Likert scale of 4 points. Cyberaddiction, sleep and eating disturbance scores were determined by adding points.

### Data analysis

The final raw data were downloaded from Google Forms and converted into a Comma Separated Value (CSV) file for analysis using SPSS software (version 21.0 for Windows). The analysis of descriptive statistics was conducted to illustrate the demographic and others elected characteristics of the respondents. A univariate analysis was used to explore the significant associations between sample characteristics and the parental anxiety level during the COVID-19 epidemic. Variables with *p* < 0.2 in univariate analysis were screened and included in the multivariate logistic regression analyses. The estimates of the strengths of associations were demonstrated by the odds ratio (OR) with a 95% confidence interval (CI). Two-tailed *p* < 0.05 was considered statistically significant.

### Ethical considerations

The current study was carried out in accordance with the ethical principles of the Declaration of Helsinki. An approval from the Institutional Review Board of the Military Hospital of Tunis was obtained (#68/2020/CLPP). All participants voluntarily gave their informed consent (within the Google form) to access the questionnaire and to participate in the study after being informed about the purpose of the study. The questionnaires were anonymous to ensure confidentiality and data reliability.

## Results

A total of 541 participants responded and completed the online survey. Three participants were excluded since they provided incomplete information.

### Characteristics of the study population

The study of the family status revealed a majority for mothers (86.2%) that were enrolled in this study (Table 1). Almost half (55.7%) of the mothers were aged between 30 and 40 years old. Most of the parents reported previous high school which accounted for 81.9% of the total population questioned. Seventy two percent of the participants were from the middle socioeconomic class with more than the half (57.1%) being employed. The respondents lived mainly inside Tunisia (98%) within urban areas (95.5%). Most of the parents had two children under 6 years of age or aged 6 through 12.

**Table 1:** Parents socio-demographic characteristics

Characteristics	Effectives (n)	Percentage (%)
**Gender**		
Male	464	13.8
Female	74	86.2
**Age (years)**		
< 25	9	1.7
25—30	51	9.5
30—35	133	24.7
35—40	167	31.0
40—45	127	23.0
Over 45	51	9.5
**Parental situation**		
Single parent	22	4.1
Married	486	90.3
Divorced	27	5
Widow	3	0.6
**Occupation**		
HCWs	21	3.9
Employed	307	57.1
Private sector workers	71	13.2
Day laborer	9	1.7
Unemployed	124	23
Student	5	0.9
Retired	1	0.2
**Educational level**		
Primary	5	0.9
High school	91	16.9
Upper/university	442	81.9
**Socio-economic level**		
Low	10	1.9
Middle	389	72
High	128	23.7
**Place of residence**		
Tunisia	436	97.8
Foreign country	10	2.2
**Residency type**		
Urban	516	95.9
Rural	22	4.1
**Number of. Children**		
0	2	0.4
1	135	25.1
2	262	48.7
3	112	20.8
4	22	4.1
Over 4	5	0.9

*HCWs* Healthcare workers

Children with a psychiatric history was reported in 15.6% of the population (Table 2). The most common diagnoses were attention-deficit/hyperactivity disorder (ADHD, 9.9%), autism spectrum disorder (ASD, 3.2%), and depression (1.3%). Somatic history accounted for 12.6% with 8.6% suffered from asthma, 1.3% epilepsy, 1.3% immunodeficiency, 0.6% deafness, and 0.4% genetic disease.

**Table 2:** Children’s socio-demographic and clinical characteristics

*Variables*	*Effectives (n)*	*Percentages (%)*
**Age (years)**		
< 6	292	42.5
6–12	294	42.7
12–18	101	14.7
**Past psychiatric history**		
None	456	84.4
ADHD	53	9.9
ASD	17	3.2
Depression	7	1.3
Mental retardation	5	0.9
**Past somatic history**		
None	471	87.2
Diabetes	2	0.4
Asthma	46	8.6
Epilepsy	7	1.3
Immunodeficiency	7	1.3
Deafness	3	0.6
Genetic disease	2	0.4

*ADHD* Attention deficit hyperactivity disorder*, ASD* Autism Spectrum Disorders

### Effects of the COVID crisis on parent and child psychological aspects

The sample average for generalized anxiety in adults accounted for 11 indicating a moderate anxiety. The level of anxiety was severe for 27.8%, moderate for 40.8%, mild for 19.2% and minimal for 12.2% of the parents (Table 3). A state for burnout was confirmed in 19.5% of cases. The proportions of parents with high, moderate, and low burnout were 13.3%, 13%, and 16.8%, respectively. It was found that 37.3% of the population did not experience a state of mental exhaustion.

**Table 3:** Parents and children psychological impact

Score	Effectives (n)	Percentages (%)
**GAD-7 score**		
Minimal	64	12.2
Mild	101	19.2
Moderate	214	40.8
Severe	146	27.8
**PBA score**		
No burnout	193	37.3
Low-risk	87	16.8
Moderate-risk	67	13
High-risk	69	13.3
Burnout	101	19.5
**Infantile anxiety score**		
No anxiety symptoms	16	2.9
Minimal	89	16.4
Mild	304	56.2
Moderate	125	23.1
Severe	7	1.3

*GAD* Generalized anxiety disorder*, PBA* Parental burnout assessment

The average for infantile anxiety score was 34 in children indicating mild anxiety behavior. The level of anxiety was severe (1.3%), moderate (23.1%), mild (56.2%), and minimal (16.4%).

### Factors associated with symptoms of anxiety among parents during the COVID-19 epidemic

Gender, occupation, and PBA were strongly associated (*p* < 0.001) with anxiety symptoms. However, age, parental situation, educational level socio-economic level, place of residence, number of children had no significant (Table 4). As for factors related to children and associated to parental anxiety, we found that the infantile trait-anxiety scale, children’s cyber addiction score, sleep disturbance score and eating disturbance score were associated with parental anxiety.

**Table 4:** Univariate analysis of variables related to parental anxiety

Variables	Anxiety score (GAD-7 score)		p
	**Minimal or mild (*n* = 165):n (%)**	**Moderate or severe (*n* = 360):n (%)**	
**Gender**			
Male	36 (50)	36 (50)	< 0.001
Female	129 (28,5)	324 (71,5)	
**Age**			
< 35 y.o	61 (32.6)	126 (67.4)	0.695
≥ 35 y.o	104 (30.8)	234 (69.2)	
**Educational Level**			
Secondary school or below	21 (23.6)	68 (76.4)	0.214
University	144 (33)	292 (67)	
**Residency type**			
Rural	7 (33.3)	14 (66.7)	0.84
Urban	158 (31.3)	346 (68.7)	
**Parental situation**			
Two parent family	150 (31.5)	326 (68.5)	0.897
Single parent family	15 (30.6)	34 (69.4)	
**Occupation**			
Unemployed	25 (21)	94 (79)	0.005
Employed	140 (34.5)	266 (65.5)	
**Socio-economic level**			
Poor	114 (29.3)	275 (70.7)	0.214
Good	44 (35.2)	81 (64.8)	
**Number of children**			
< 2 children	122 (31.3)	268 (68.7)	0.902
> 2 children	43 (31.9)	92 (68.1)	
**PBA**			
Minimal or mild	138 (38)	225 (62)	
Moderate or severe	17 (13.9)	105 (86.1)	< 0.001
Somatic history of children			
Yes	146 (31.8)	313 (68.2)	0.621
No	19 (28.8)	47 (71.2)	
Psychiatric history in children			
Yes	142 (32)	302 (68)	0.522
No	23 (28.4)	58 (71.6)	
The infantile trait-anxiety scale			
Minimal or mild	138 (83.6)	27 (16.4)	< 0.001
Moderate or severe	206 (57.2)	154 (42.8)	
Children’s cyberaddiction score			
Median [Q25%-Q75%]	6 [4,–9]	8.5 [5,–10]	< 0.000
Children’s eating disturbance score			
Median [Q25%-Q75%]	1 [0–2]	2 [1,–3]	< 0.000
Children’s sleep disturbance score			
Median [Q25%-Q75%]	2 [0.5–3.5]	3 [1.5–5]	< 0.000

*GAD* Generalized anxiety disorder*, PBA* Parental Burnout Assessment

To eliminate confounding factors and determine the variables independently associated with parental anxiety symptoms, a multivariate regression model was used. The results revealed four determining factors. Occupation was identified as a protective factor against anxiety. Whereas parents with moderate or severe burnout were significantly at higher risk of being anxious (OR = 2.834). In addition, the study showed that the parental anxiety score increased significantly among parents whose children have higher levels of cyber addiction and eating disorders (Table 5).

**Table 5:** Multivariate analysis of variables related to parental anxiety

Variables	*OR/Beta*	*CI*_*95%*_	*p*
Occupation (OR)	0.516	[0.30–0.86]	0.012
Parental Burnout Assessment (OR)	2.834	[1.74–4.60]	< 0.000
Cyber addiction score (Beta)	1,099	[1.02–1.17]	0.005
Eating disturbance score (Beta)	1.248	[1.07–1.45]	0.004

*OR* Odds Ratio, *CI*_*95%*_ Confidence Interval 95%

## Discussion

The SARS-COV-2 epidemic has profoundly affected Tunisian families. The impact of prolonged isolation and confinement at home are causing profound changes in family routines and habits. The governmental decision to postpone school activities and lock up children at home was maybe necessary, but the consequences on the mental health of families were barely considered.

This study was initiated to examine the impact of the COVID-19 epidemic on the psychological and emotional health of parents and children of a Tunisian-North African population. More than half of the parents reported moderate to severe level of anxiety and children’s anxiety was positively associated to their parent’s with one in four of children having evidence of moderate anxiety.

In the current pandemic context, some earlier studies have also shown that children’s assessments were correlated with those of their parents, suggesting the important link between the mental health of parents and their children [17–19]. Parenting can be stressful under normative circumstances like confinement [20–23].

Many families are isolated at home under the influence of an accumulated stress and pressure. They are unable to receive support from the extended family and could no longer engage in leisure activities, which can exacerbate the impact of these stressors [24]. In this study, mothers had higher levels of anxiety resulting from the pandemic COVID-19 confinement. This result is consistent with previous studies evaluating the psychological impact of COVID-19 on parents and reporting that mothers were more severely affected [25–27]. Our study and earlier ones call for psychosocial attention to be paid to women.

Although the confinement during the COVID-19 pandemic resulted in beneficial increasing responsibilities for parents, families were expected to report financial difficulties as an additional familial stress factor during the pandemic. This study revealed a significant association between discontinued professional activity and anxiety symptoms which suggest that financial difficulties may trigger psychological issues like anxiety. Juan et al. suggested that financial loss, resulting from midlife, is a risk factor for symptoms of psychological disorders, anger, and anxiety [28].

Parental burnout and its risk factors were elevated in participants of the current study following the pandemic COVID19. During this specific period, parental unemployment, financial insecurity, and lack of free time may trigger parental emotional and mental exhaustion and increase the risk of burnout as previously reported [29]. These changes may increase parental risk of burnout as they struggle to find time for themselves and balance their personal, work and parental responsibilities. While many parents have lost their jobs, many others have switched to working from home while involving their children in home schooling or virtual education activities.

The current pandemic situation is particularly unique since access to traditional social supports from extended family and friends has been cancelled or very limited. Many parents often rely on the support of children’s grandparents or other family members for childcare or assistance with parenting activities. An important number of studies evidenced the importance of social supports for parental well-being, and contact with grandparents, can reduce the risk of parental burnout [30]. In addition, it was reported an association between parental burnout and child abuse and neglect [31, 32]. These associations have also been supported by intervention research, which has shown that child abuse and neglect can be reduced by interventions aimed at decreasing levels of parental burnout [33].

During this special period, the psychological impact is experienced both at the individual level (feeling anxious and irritated) and at the dyadic level (having difficulties to interact with the child). However, little was known about the psychological distress of children exposed to the pandemic. Although, children may be particularly vulnerable to the psychological effects of COVID-19 [6, 8, 34, 35]. It has been postulated that fear can be contagious and that children are extremely sensitive to the emotional state of the adults around them who constitute their first source of emotional security. Thus, it can be particularly frightening for a child to perceive that one of or both parents are in distress and unable to prevent an occurring traumatic event. In addition, the more stressed are the parents, the more they experience difficulty in understanding and responding sensitively to their child's needs [16]. In addition, children have fewer personal resources to cope with the pandemic consequences [36]. The role of the parents was particularly important in mitigating the psychological effects of confinement. Child developmental studies highlighted that children rely on trusted adults, in particular their parents, for protection and referencing for danger assessment and making sense of the events [37]. Besides, anxiety is often associated with rude behavior and difficulties in explaining boundaries and discipline. As a result, children may feel less understood by their parents and may react more negatively and aggressively [38]. Parents may interpret this as misbehavior, oppositional behavior and tantrums.

During the lockdown period, schools were closed and children were forced to stay at home. Whereas, a recent study suggested that when children are not attending the school, they are less physically active, have poorer sleep hygiene, and spend more time in front of screens [39]. In these times of severe social isolation, the amount of time that children and adolescents spend in front of the screen has increased dramatically. In addition, the overuse of social networks makes them vulnerable to online predators, cyber bullying and potentially harmful content. Besides, disturbances resulting from the current pandemic were associated with behavioral problems [40, 41]. These behaviors can lead to parental doubts and feelings of inadequacy and difficulties in understanding and empathy, increasing feelings of sadness and lack of control, lack of sleep, and lack of attention from caregivers. This supports the results of this study suggesting that children's life habits during the lockdown are linked to parental anxiety.

These results suggest many implications that should be addressed in the short and long-term in all countries, affected by the pandemic. Parents should maintain, in a comfortable family environment and adequate sleep, the children's daily rhythms of life, such as balance between work and rest, regular activities and entertainment. Other studies are required to study the medium and long-term consequences of the pandemic in all countries affected by the virus, particularly Tunisia. Interventions designed to promote positive adaptations to the COVID-19 crisis should be aimed main at improving family financial conditions, providing adequate childcare, and increasing job security and flexibility for parents, as these interventions can help improving the mental status of children. For children, it is suggested to educate them, maintain safe distances from screens, practicing personal hygiene.

Some limitations of the present study should be addressed. First, this is a correlational modality, and the recall period was relatively short. A longitudinal exploration of the effects of quarantine on parents and the cascading effects on children over time can help a better understand of the phenomenon. Second, the sample was self-selected, and the population was limited and targeted. Third, our data rely on self-reports rather than clinical health assessments, and without other measures of past psychopathology. Finally, we have collected children’s psychological symptoms from parent reports; although this data collection method may be less informative than direct evaluation of children’s well-being made by experts.

## Conclusion

This study was done during the mandatory lockdown during the first wave of Corona (6 weeks) to assess the impact of the COVID-19 lockdown on parents and children on psychological well-being. Its findings highlight the severe psychological impact of the confinement on parents with more than half of parents reported moderate to severe level of anxiety and with 19.5% affected by a burnout. Results also show the close link between parent and child distress. These findings raise serious concerns about the need for specific social and psychological support programs for parents and families, both during and after the pandemic. Further research is required to assess pandemic short and long-term consequences of covid-19 pandemic.

## Data Availability

The data that support the findings of this study are not publicly available. Data are, however, available from the corresponding author upon reasonable request.
